# Exploring Bioimage Synthesis and Detection via Generative Adversarial Networks: A Multi-Faceted Case Study

**DOI:** 10.3390/jimaging11070214

**Published:** 2025-06-27

**Authors:** Valeria Sorgente, Dante Biagiucci, Mario Cesarelli, Luca Brunese, Antonella Santone, Fabio Martinelli, Francesco Mercaldo

**Affiliations:** 1 Department of Medicine and Health Sciences “Vincenzo Tiberio”, University of Molise, 86100 Campobasso, Italy; d.biagiucci@studenti.unimol.it (D.B.); luca.brunese@unimol.it (L.B.); antonella.santone@unimol.it (A.S.); 2 Department of Engineering, University of Sannio, 82100 Benevento, Italy; mcesarelli@unisannio.it; 3 Institute for High Performance Computing and Networking, National Research Council of Italy, 87036 Rende, Italy; fabio.martinelli@icar.cnr.it

**Keywords:** GAN, convolutional generative adversarial network, DCGAN, deep convolutional generative adversarial network, deep learning, bioimages, classification

## Abstract

Background:Generative Adversarial Networks (GANs), thanks to their great versatility, have a plethora of applications in biomedical imaging with the goal of simulating complex pathological conditions and creating clinical data used for training advanced machine learning models. The ability to generate high-quality synthetic clinical data not only addresses issues related to the scarcity of annotated bioimages but also supports the continuous improvement of diagnostic tools. Method: We propose a two-step method aimed to detect whether a bioimage can be considered fake or real. The first step is related to bioimage generation using a Deep Convolutional GAN, while the second step involves the training and testing of a set of machine learning models aimed to distinguish between real and generated bioimages. Results: We evaluate our approach by exploiting six different datasets. We observe notable results, demonstrating the ability of Deep Convolutional GAN to generate realistic synthetic images for some specific bioimages. However, for other bioimages, the accuracy does not align with the expected trend, indicating challenges in generating images that closely resemble real ones. Conclusions: This study highlights both the potential and limitations of GAN in generating realistic bioimages. Future work will focus on improving generation quality and detection accuracy across different datasets.

## 1. Introduction

In the last few years, Artificial Intelligence (AI) [1] has revolutionized biomedical research, enabling unprecedented technological leaps. As a matter of fact, AI-powered approaches have not only enhanced the efficiency of data analysis but also opened new possibilities for tackling complex medical challenges, from disease diagnosis to personalized treatment planning. These advancements have profoundly impacted healthcare, driving the transition from traditional methods to data-driven, AI-enabled innovations.

Among the myriad of different that AI enables, Generative Adversarial Network (GAN) has emerged as a set of algorithms devoted to image generation, particularly in the biomedical field [2,3]. GANs consist of two neural networks, i.e., the generator and the discriminator, that work in tandem to create synthetic data that, possibly, are indistinguishable from real-world ones [4,5]. This adversarial learning framework has demonstrated remarkable potential in various applications, including the generation of synthetic medical images, enhancement of data for rare diseases, and improvement of AI model performance in clinical settings.

With particular regard to biomedical research, a well-known challenge in the community is the scarcity of high-quality labeled bioimages. The preparation of such datasets is often labor-intensive, time-consuming, and constrained by privacy regulations, leading to a bottleneck in the development and training of deep learning models. GANs can address this issue by generating synthetic bioimages, which serve as a powerful augmentation strategy for expanding datasets in size. This can enable researchers to train AI models with diverse and representative samples, improving their accuracy and generalizability. Furthermore, GANs can be utilized for critical image processing tasks such as noise reduction, artifact removal, and resolution enhancement, all of which contribute to the production of clearer, clinically valuable images for diagnostic purposes.

Beyond data augmentation and noise reduction, GANs have also been explored for automating the segmentation of organs and tissues. Such applications simplify the identification of structures of clinical interest, thereby aiding in disease diagnosis, surgical planning, and treatment monitoring. For instance, the ability of GANs to simulate complex pathological conditions enables researchers to study diseases in greater detail and to create virtual datasets for rare or hard-to-obtain scenarios.

Despite their generative capabilities, GANs come with limitations and challenges [6]. A key concern is the introduction of biases in synthetic data, which can arise from imbalanced or unrepresentative training datasets. These biases can inadvertently propagate into downstream models, potentially compromising their reliability and fairness in real-world clinical applications. Moreover, ethical and legal challenges associated with GANs include the privacy and ownership of synthetic data. The possibility of synthetic data being misused for unauthorized purposes raises questions about intellectual property, data protection, and patient consent, necessitating the establishment of comprehensive ethical guidelines and governance frameworks.

Nonetheless, the potential of GANs to revolutionize biomedical research is undeniable. By simulating realistic pathological conditions and generating synthetic datasets, GANs enable researchers to overcome data scarcity and improve the precision of AI models. This, in turn, enhances the reliability and scalability of diagnostic tools, paving the way for transformative healthcare solutions.

Starting from these considerations, in this paper, we propose a method aimed to distinguish between real and fake bioimages by exploiting machine learning. To evaluate the proposed method on a set of different kinds of bioimages, we consider real bioimages from six diverse datasets encompassing common diseases, while synthetic images are generated using a GAN. In detail, for synthetic bioimage generation, a Deep Convolutional Generative Adversarial Network (DCGAN) is utilized. We consider the DCGAN because it is particularly suited for biomedical applications due to its ability to produce high-quality, realistic images that preserve critical clinical features. Its architecture is optimized for learning complex patterns, making it a valuable tool for advancing both research and clinical applications in the biomedical domain.

To evaluate the proposed method in the experimental analysis, we compute and present the results of a set of metrics, i.e., *Precision*, Recall, F-measure, and Accuracy, aimed to assess the effectiveness and robustness of the discrimination between fake and real bioimages.

We aim to provide insights into the capabilities and limitations of GANs in generating synthetic medical images and their implications for biomedical research. The findings could contribute to a deeper understanding of how synthetic data can be effectively integrated into clinical workflows, ultimately supporting the development of more robust and precise AI-driven healthcare solutions.

The paper proceeds as follows: in the next section, we present an overview of the state-of-the-art in the context of the adoption of GAN in biomedical research, Section 3 presents and describes the proposed method aimed to discriminate between fake and real bioimages, in Section 4, the results of the experimental analysis are discussed for each case study, and finally, in the last section, we draw a conclusion and future research plans.

## 2. Related Work

There is a growing body of research in the literature where GANs are applied in the biomedical field for a wide range of purposes and objectives [7]. These studies explore various aspects of medical imaging, data augmentation, and disease modeling, aiming to improve diagnostic accuracy, enhance training datasets, and simulate complex pathological conditions that are often difficult to capture using traditional methods. For example, Orlando et al. [8] introduced a technique for generating retinal fundus images with simulated lesions, aiming to enhance diagnostic models, particularly in the early detection of retinal diseases. In another work, Fu et al. [9] employed GANs to augment retinal fundus image datasets.

These interesting applications of GANs serve not only to improve the performance of diagnostic tools but also to expand the potential for more precise and personalized medical care.

As a matter of fact, GANs have demonstrated significant potential in the biomedical field [2], with applications ranging from medical image synthesis to clinical data simulation. A key challenge in clinical practice is the limited availability of high-quality labeled biomedical images required for training deep learning models in diagnostic tasks. GANs address this issue by generating realistic synthetic medical images, thereby expanding datasets and enhancing model robustness. However, the adoption of GANs introduces certain risks, including the potential for fraudulent use. For instance, GANs could be exploited to create falsified diagnostic images, such as X-rays or Magnetic Resonance Imaging (MRIs), with the intent to manipulate diagnoses. Additionally, the generation of altered synthetic data could undermine the validity of clinical or epidemiological studies. These risks highlight the importance of ethical considerations and the need for strategies to safeguard the integrity and reliability of biomedical data.

Several studies have explored the use of GAN-based synthetic image generation to enhance classification performance in medical imaging. For example, Frid-Adar et al. [10] focused on liver lesion classification by generating diverse synthetic lesion images using GANs to address class imbalance and limited data availability. By incorporating these synthetic images into the training set, they achieve notable improvements in CNN classifier sensitivity and specificity, demonstrating enhanced robustness in lesion detection and differentiation between lesion types.

Despite their potential, GANs face several challenges that limit their effectiveness, such as difficulties in training and issues like mode collapse, which hinder their ability to generate diverse and accurate outputs. As a matter of fact, numerous studies have investigated the application of GANs, considering also this aspect, in the biomedical domain for various purposes [7,11,12,13].

In particular, Kufel et al. [14] provided a comprehensive review of AI in medicine, highlighting the growing use of GANs to generate synthetic data such as computed tomography (CT) scans, MRIs, mammograms, and electrocardiograms (ECGs). These data augment the training sets and enable the modeling of rare pathologies. The authors also discussed hybrid architectures where GANs are integrated with autoencoders to improve feature encoding and reconstruction, particularly for anomaly detection tasks.

Beyond generation, recent literature has emphasized the importance of detecting GAN-generated medical content. As GANs become more sophisticated, distinguishing synthetic from real images becomes increasingly challenging, with implications for data integrity, clinical safety, and medical forensics. This is particularly relevant in radiology and pathology, where highly realistic synthetic images may mislead automated diagnostic systems. Several open-source benchmarks have emerged to address this issue, inspired by initiatives such as the DeepFake Detection Challenge (DFDC), originally developed for facial image forgery. While DFDC focuses on multi-media content, it has inspired similar research in the medical domain, aimed at developing classifiers capable of identifying synthetic diagnostic data. These benchmarks highlight the need to codevelop detection systems alongside generative models to ensure the safe deployment of GANs in healthcare.

Emerging from this discussion, GANs have been employed for tasks such as retinal vessel segmentation and liver lesion classification, while this study focuses on generating synthetic bioimages that closely mimic real ones, with the aim to understand if bioimages GAN-generated can be discriminated from real ones. We hypothesize that as the number of training epochs increases, the quality of synthetic images will improve, making them progressively more realistic and harder for classifiers to distinguish from genuine samples. This trend underscores both the transformative potential of GANs for realistic image synthesis and the challenges they pose to current diagnostic and classification systems. We consider a multi-faceted case study, composed of six different experimental analyses performed on different bioimages with the aim to evaluate the proposed method of different bioimage scenarios.

## 3. The Method

In the following section, we present and describe in detail the proposed method for fake bioimage detection. We also describe the six datasets utilized for the several case studies we proposed to assess the experimental analysis results.

**Figure 1:**
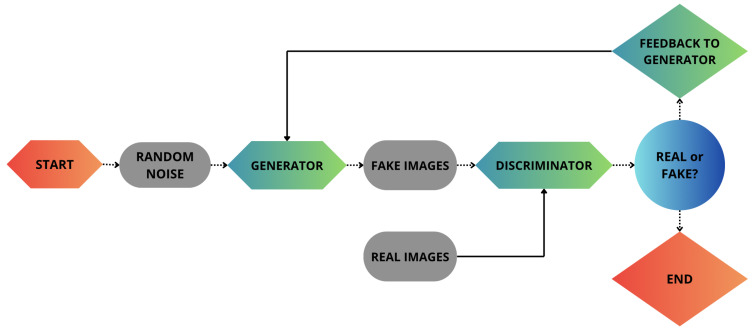
The first step of the proposed method involves the generation of synthetic bioimages.

**Figure 2:**
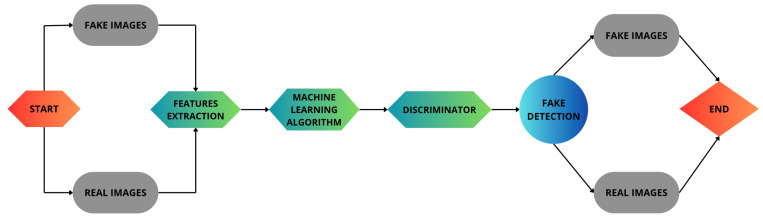
The second step of the proposed methodology involves the detection of synthetic bioimages.

The proposed approach is structured in two different phases. The first phase focuses on the generation of fake bioimages using GANs, as shown in Figure 1. In the second phase, we build a set of machine learning models aimed to compare the real bioimages with respect to the fake ones produced in the previous step, as shown in Figure 2. In the second step, we evaluate the accuracy and quality of the generated bioimages.

As shown in Figure 1, GANs consist of two competing neural networks: the generator and the discriminator. Specifically, the generator creates synthetic images, while the discriminator attempts to distinguish them from real images. This process iterates until the generator produces data indistinguishable from real ones.

To do this, it is necessary to find parameters for the discriminator that maximize its accuracy, and at the same time those of the generator that are able to confuse the discriminator as much as possible.

As shown in Figure 1, the generator cannot access real images directly, but learns only through its interaction with the discriminator. Instead, the latter analyzes both types of images, real and generated, and is trained to distinguish between them. This process causes the generator to progressively improve and generate increasingly realistic images.

Both the generator and the discriminator are typically made up of multi-layer neural networks, which in turn are made up of convolutional or fully connected layers. The generator network can be defined as the mapping of points from the latent space to a real one. In practice, it transforms a set of abstract features into real data. The discriminator network, on the other hand, is usually a binary classifier that evaluates the probability of each image to be synthetic or real, assigning values between 0 and 1, respectively.

In our work, we implemented a DCGAN architecture, following the model introduced by Radford et al. [15]. The generator receives a 100-dimensional noise vector and processes it through a Dense layer (12,544 units), followed by Batch Normalization and ReLU activation. The output is reshaped into a 7 × 7 × 256 tensor and then upsampled using two Conv2DTranspose layers (with 128 and 64 filters), each followed by Batch Normalization and ReLU. Finally, a Conv2D layer with one filter and linear activation produces 28 × 28 grayscale images. The discriminator is composed of two Conv2D layers (with 64 and 128 filters), both followed by Batch Normalization and LeakyReLU. After flattening, a Dropout layer is applied, and the final Dense layer with sigmoid activation outputs the classification score (real or fake).

By fixing the generator, it is possible to train the discriminator to distinguish the images. Once this process has been completed, the generator is trained, in order to reduce the accuracy of the discriminator.

In the following, we describe in more detail how the proposed DCGAN works.

The generator model expects input images with dimensions (28, 28), indicating that the training data consists of grayscale images of size 28×28. The images are, therefore, single-channel.

To ensure compatibility with the generator’s output layer, which uses a tanh activation, we normalize the pixel values of the images to the range [−1,1].

The primary purpose of the generator is to synthesize realistic images that can successfully deceive the discriminator into classifying them as real.

The generator takes a vector of random noise as input and produces an output image that closely resembles those from the training set. Since we are generating grayscale images of dimensions 28×28, the output shape of the generator must be (28,28,1).

To achieve this, the generator follows the steps below:

1. It transforms the 1D random noise (latent vector) into a 3D shape using a Reshape layer;
2. It upscales the intermediate features using Conv2DTranspose layers (also known as fractional-strided convolutions) until the output size of 28×28×1 is obtained.

The generator includes several key layers:

1. Dense: Used to expand and reshape the latent vector;
2. Conv2DTranspose: Employed for upsampling to gradually increase the image resolution;
3. BatchNormalization: Added after convolutional layers to stabilize training and accelerate convergence.

All intermediate layers use ReLU activation, while the final output layer uses tanh.

A function is used to construct the generator model, and its architecture is summarized in Table 1.

The generator is implemented using Keras’ Sequential API. The initial Dense layer expands the input noise and reshapes it to a 3D format. Then, two upsampling stages are applied using Conv2DTranspose layers with stride 2, transitioning from 7×7 to 14×14, and finally to 28×28.

Each Conv2DTranspose layer is followed by a BatchNormalization and ReLU activation. The final output layer is a Conv2D layer with a tanh activation.

The total number of parameters in the generator is 2,343,681, with 2,318,209 being trainable and 25,472 non-trainable.

Next, we define the discriminator model.

The discriminator is a binary classifier that aims to distinguish between real and fake images. Its main objective is to correctly classify real images (label = 1) and fake images generated by the generator (label = 0).

Key differences between the discriminator and a traditional classifier include:

1. Use of LeakyReLU instead of ReLU to avoid dying neurons;
2. Dual input nature: real images from the dataset and fake images from the generator.

The discriminator is intentionally kept relatively simple to avoid overpowering the generator and hindering its learning.

Table 2 summarizes the architecture of the discriminator.

The discriminator model accepts input images of shape (28,28,1). It includes two blocks consisting of Conv2D, BatchNormalization, and LeakyReLU layers. After feature extraction, the output is flattened, a dropout is applied, and a Dense layer with sigmoid activation returns a single probability value.

The discriminator comprises a total of 213,633 parameters, of which 213,249 are trainable and 384 are non-trainable.

Loss computation is a fundamental component of DCGAN training.

We utilize the modified minimax loss, implemented via the Binary Cross-Entropy (BCE) loss function. Two losses must be calculated:

Discriminator Loss: Since the discriminator handles both real and fake samples, we compute separate losses for each and sum them:

(1) L∗D=BCE(D(x∗real),1)+BCE(D(G(z)),0)

Generator Loss: Instead of minimizing log(1−D(G(z))), we train the generator to maximize logD(G(z)), encouraging the generator to produce images that the discriminator classifies as real:

(2) L_G=BCE(D(G(z)),1)

In short, the parameters of each model are alternatively set as fixed, in order to train those of the other model. The generator is considered perfectly trained when the probability of an image being real equals that of being artificially generated, for all samples of the dataset provided.

This represents an ideal practice since the discriminator is not always trained to its maximum performance, but only for a limited number of iterations, and the generator and discriminator are not always trained alternately but can do so simultaneously.

In the current study, training was performed using Binary Cross-Entropy loss and the Adam optimizer (learning rate = 0.0002, $\beta_{1}=0.5$), which is a widely used setup to improve training stability [16]. During training, the generator is updated to “fool” the discriminator, while the discriminator is optimized to correctly distinguish real from fake samples. This adversarial process pushes the system toward an equilibrium where fake images become indistinguishable from real ones [17].

Figure 2 shows the second step of the proposed method, i.e., the fake bioimage detection one. This step begins with two input categories: real bioimages and fake bioimages generated by GANs. These images are directed into a feature extraction stage, where key characteristics of the images are identified and processed. The extracted features are then fed into a machine learning algorithm, which learns to differentiate between real and fake images.

Following this, the discriminator plays a crucial role by analyzing the outputs of the machine learning model and classifying the images as real or fake. The results from the discriminator flow into the fake detection module, which computes a set of metrics aimed to assess the effectiveness of the proposed method.

## 4. Experimental Analysis

In this section, we present the experimental analysis, conducted to evaluate the effectiveness of the proposed method. The analysis was designed to thoroughly assess the performance and demonstrate how well the method achieves its objectives under different scenarios.

### 4.1. Datasets

To evaluate the proposed method, we resort to the MedMNIST [18] collection, a repository of biomedical image classification resources, freely available for research purposes.

In detail, the datasets analyzed in this study are as follows:

- Blood_MNIST, which is obtained from blood tests conducted using a blood cell microscope;
- Breast_MNIST, which contains images inherent in mammography examinations using ultrasound methodology;
- Tissue_MNIST, which includes histological images acquired by a kidney cortex microscope;
- Organ_SMNIST, Organ_CMNIST, Organ_AMNIST, which contain images of different human organs collected by abdominal CT.

For each of the six datasets, 1000 random images with dimensions of 28 × 28 are considered.

### 4.2. Experimental Settings

For image generation, we considered a Python script developed from authors. For each one of the six dataset, 1000 real bioimages are considered. We ran the GAN for six times, each time with a different dataset. Each time, the GAN was run for 50 epochs and for each epoch, 1000 bioimages were generated.

Therefore, for each case study, 1000 images were generated in each of the 50 epochs, for a total of 50,000 images for each case study and 300,000 images overall for the entire experimental analysis.

**Figure 3:**
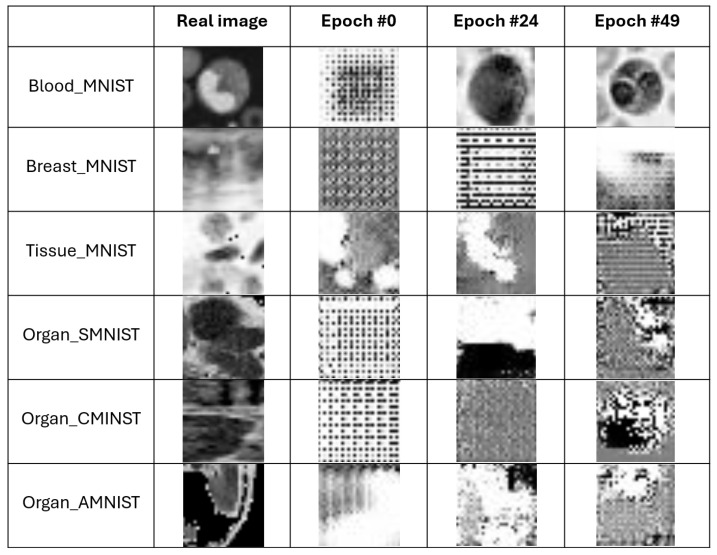
Visual representation of a set of images generated by DCGAN in different epochs i.e., the first (#0 epoch), the middle (#24 epoch) and the last epoch (#49 epoch).

For example, in Figure 3 we show a set of images generated by the exploited DCGAN. Specifically, each row is related to a different dataset and, in each row, the first image corresponds to the original image, while the subsequent images showcase the ones generated at epochs 0, 24, and 49, respectively, highlighting the evolution of the model’s output over these different training stages.

In order to discriminate real bioimages from generated ones, it is necessary to extract numerical features that could be used to build a machine learning model. At this stage, the images underwent a preprocessing phase during which different filters were applied to emphasize chromatic differences of the individual pixels in each image.

Specifically, the three filters used are as follows:

- JPegCoefficentFilter, which is a feature extraction method that captures frequency-domain information by analyzing the Discrete Cosine Transform (DCT) coefficients embedded in JPEG-compressed images. These coefficients, which form the basis of JPEG compression, reflect the texture and structural characteristics of the image, making the filter useful for tasks such as image quality assessment or compression-based pattern recognition.
- AutoColorCorrelogramFilter, which is a descriptor that measures how colors are spatially distributed across an image by computing the probability of finding a pixel of the same color at a specific distance from another pixel. This feature effectively combines color information with spatial layout, providing a richer representation for scene classification and image retrieval applications.
- ColourLayoutFilter, inspired by MPEG-7 standards, extracts a compact representation of the spatial arrangement of colors in an image. It divides the image into a grid, determines the dominant color in each block (typically using YCbCr color space), and applies a DCT transformation to encode the layout. This allows for a perceptually meaningful and highly compressed representation of color distribution.

Preprocessed images were classified using four classification algorithms, J48, REPTree, Random Forest, and LMT [19].

J48 is the Weka implementation of the C4.5 algorithm, which generates decision trees by recursively partitioning data based on the attribute that yields the highest information gain. It is widely used due to its interpretability and support for both numeric and nominal data.

The RepTree classifier is another decision tree method that focuses on speed and simplicity. It builds trees using information gain and prunes them using reduced-error pruning, often resulting in faster training and simpler models, although potentially at the cost of reduced accuracy in some scenarios.

Random Forest is an ensemble learning technique that constructs a collection of decision trees during training and outputs the class that represents the majority vote of the individual trees. By averaging the predictions of multiple diverse models, it significantly improves generalization performance and reduces overfitting, making it suitable for complex and high-dimensional datasets. In this study, the Random Forest classifier achieved the best performance.

Finally, LMT, or Logistic Model Tree, is a hybrid classifier that merges decision tree structures with logistic regression at the leaves. Instead of assigning a fixed class at each leaf node, LMT fits a logistic regression model, allowing for more nuanced decision boundaries. This combination enables LMT to capture both non-linear relationships through the tree structure and linear separations via logistic regression, often resulting in high classification accuracy with enhanced interpretability.

Thus, for each one of the six different case studies, we perform 50 classifications with each of the considered three filters and four models: each classification is conducted with the 1000 real images of the dataset and with the 1000 images generated for a given epoch. This is the reason why the total number of classifications conducted is number of algorithms (i.e., 4) * number of filters (i.e., 3) * number of epochs (i.e., 50) = 600 classifications for a case study. Considering that in the experimental analysis, we consider six different case studies, we have a total number of classifications equal to 600 * number of datasets (i.e., 6) = 3600, i.e., the number of the different classifications that have been considered in the experimental analysis.

By combining image filters and classifiers, a thorough analysis of metrics was conducted to evaluate the realism of the images generated by DCGAN.

As a matter of fact, we expect that the accuracy of the various classifiers will decrease as the epochs increase, since DGCAN is expected to generate images that are closer to the real ones as the epochs increase and, therefore, the classifier will be less effective in discriminating the images.

### 4.3. Experimental Results

Several metrics were computed with the aim to evaluate the performance of the classification between real and fake GAN-generated bioimages; in particular, the following metrics are considered:

- Precision is a measure of correctly classified instances of the total number of instances in the dataset.
  (3) $$Precision=\frac{TP}{TP+FP}$$
  where TP (True Positive) represents the number of instances correctly identified as positive while FP (False Positive) is the number of negative instances misclassified as positive.
- Recall is a measure of completeness that describes the ability to correctly classify all positive instances.
  (4) $$Recall=\frac{TP}{TP+FN}$$
  where FN (False Negative) describes the number of instances misclassified as negative.
- F-Measure is the harmonic mean of Precision and Recall and it is particularly useful for evaluating the performance of classification models, especially when dealing with imbalanced datasets.
  (5) $$F\text{-}Measure=2*\frac{Precision*Recall}{Precision+Recall}$$
- Accuracy represents the measure of a model’s performance, the proportion of patterns that are correctly classified compared to all those present in the dataset.
  (6) $$Accuracy=\frac{TP+TN}{TP+TN+FP+FN}$$
  where TN (True Negative) is related to the number of instances correctly classified as negative.

Table 3 shows the average Precision, Recall, F-Measure, and Accuracy at three different epochs, i.e., the most representative ones, which are the first epoch, the 25th epoch, and the last epoch. The highest performance results for the six datasets used in the case studies, achieved with the combination of Random Forest algorithm and ColourLayout filter, were presented. Additionally, comparable levels of performance were obtained when applying the Random Forest algorithm with the other three filters previously mentioned, further demonstrating the robustness and versatility of the approach across different filter types.

The classification process produced heterogeneous results among the six datasets, both in terms of the combinations of image filters and classification algorithms used, as well as the Accuracy trend over the epochs. The results for each dataset will be discussed in the following.

It is important to highlight that the extremely high values reported across all metrics, especially in the early epochs, suggest that the visual and statistical differences between real and generated images are still significant at the beginning of training. As expected, the generator initially produces low-quality synthetic images structurally less coherent than real bioimages. This makes it easier for traditional machine learning classifiers to separate real from synthetic content, resulting in perfect or near-perfect performance.

The consistent perfect scores (Accuracy = 1.0 across epochs) in datasets like Tissue_MNIST observed a fundamental divergence between real and synthetic distributions. This aligns with the observation that handcrafted features (e.g., ColorLayout) are sensitive to low-level artifacts in early GAN outputs (e.g., checkerboard patterns, unrealistic textures). While perfect classification might seem desirable, it highlights a failure mode in generative modeling: the synthetic images are not yet clinically or biologically plausible, as they fail to mimic the complexity of real bioimages.

This trend is particularly evident in datasets like Breast_MNIST and Tissue_MNIST, where classifiers maintain ideal performance even at later epochs. Such behavior can be linked to simpler morphological features or limited intra-class variability in the original data. In contrast, datasets such as Blood_MNIST and Organ_CMNIST show a more gradual decline in classifier performance, suggesting a progressive increase in the realism of the generated images. In all cases, these metrics should be interpreted not as an artifact, but as a sign of the classifiers’ high sensitivity to subtle variations between the two image domains. Their ability to sustain high performance demonstrates their effectiveness in identifying precisely differences, when present.

**Figure 4:**
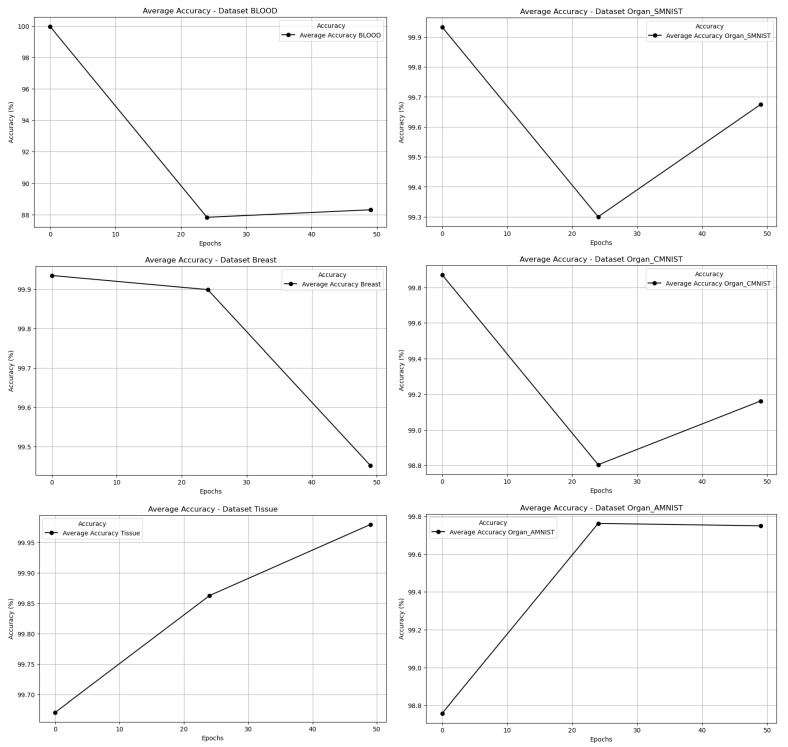
Average Accuracy while epochs are increasing for the multi-case study.

Figure 4 shows six different plots related to the average Accuracy while epochs are increasing for the multi-case study.

The first plot, corresponding to the BLOOD dataset, shows a sharp decline in accuracy after the initial epochs, eventually stabilizing at a much lower value. This suggests that while the classifier initially performs well, its ability to maintain high accuracy diminishes as training progresses. This could indicate that the synthetic images generated by the DCGAN do not sufficiently capture the complexity of the real BLOOD dataset, leading to poorer classification performance over time.

For the Organ_SMNIST dataset, the accuracy trend follows a “V” shape, with a significant drop in the middle epochs followed by a recovery toward the later stages. This pattern may indicate that the classifier struggles initially as it adapts to variations between synthetic and real images but regains performance as training progresses, possibly due to improvements in the quality of the synthetic images produced.

In the Breast dataset, the accuracy decreases steadily after an initially strong performance, implying that the synthetic images generated may not effectively represent the real image distribution. This decline could also point to overfitting or a lack of robustness in the classifier when exposed to synthetic data over time.

The Organ_CMNIST dataset exhibits a similar trend to Organ_SMNIST, with an early drop in accuracy followed by partial recovery. This pattern suggests that the classifier initially struggles to adapt to differences between synthetic and real images but manages to regain some performance as the quality of synthetic images improves or as it learns to generalize better.

For the Tissue dataset, the accuracy increases consistently across epochs, reflecting a steady improvement in the classifier’s ability to distinguish between synthetic and real images. This suggests that the synthetic images generated for this dataset are of increasingly high quality and closely resemble the real images, allowing the classifier to perform better as training continues.

Finally, the Organ_AMNIST dataset shows a nearly linear increase in accuracy, with the classifier achieving its highest performance by the end of training. This trend indicates that the synthetic images generated by the DCGAN are progressively improving in quality and becoming more representative of the real images, enabling the classifier to achieve optimal performance.

These plots highlight varying levels of success in the ability of DCGAN to generate high-quality synthetic images, with the Tissue and Organ_AMNIST datasets showing the most consistent improvements, while BLOOD and Breast datasets demonstrate challenges in maintaining classifier accuracy.

The persistent high accuracy in datasets like Breast_MNIST, where metrics remain at 1.0 even in later epochs, suggests that the DCGAN fails to close the domain gap for these specific bioimages. This could stem from the dataset’s inherent simplicity or the generator’s inability to capture critical features of real images. In contrast, the declining accuracy in Blood_MNIST or the ‘V’ shape of Organ_SMNIST reflects a partial alignment between synthetic and real distributions, though initial perfect scores still reveal stark initial differences.

**Figure 5:**
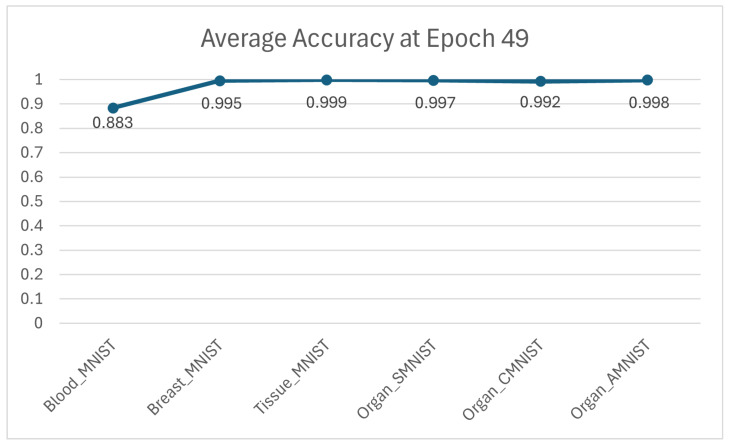
Average accuracy of the six datasets at the epoch 49.

Furthermore, as illustrated in Figure 5, the paper presents a comparative analysis of the resulting average accuracy across six distinct datasets, evaluated specifically at epoch 49, highlighting the performance variations and trends observed at the final stage of the training process.

Overall, these results demonstrate that the discriminative power of handcrafted features, when combined with classical classifiers, is highly sensitive to the degree of visual and statistical alignment between real and synthetic images. Early high classification performance indicates not a methodological flaw but the model’s effectiveness in detecting artifacts in undertrained synthetic images.

### 4.4. Critical Discussion of the Results

The trends observed across the six datasets reveal that the proposed method performs differently depending on the structural complexity and variability of the underlying bioimages. For example, datasets such as Tissue_MNIST and Organ_AMNIST show steadily increasing accuracy, indicating that the DCGAN is particularly effective at generating synthetic images that closely resemble real samples in these domains. This may be due to relatively homogeneous visual patterns in the source images, which are easier for the generator to learn.

On the other hand, datasets like Blood_MNIST and Breast_MNIST exhibit decreasing or unstable accuracy over time, suggesting that the generator struggles to reproduce the complex or noisy patterns found in those bioimages. In such cases, the visual heterogeneity and fine-grained features of real images may not be well captured by the current GAN configuration.

To address these limitations and improve model performance on more complex datasets, future work may explore alternative generative architectures such as Wasserstein GANs (WGANs) or StyleGANs, which are known to offer better training stability and detail preservation. Additionally, incorporating domain-specific image priors or using hybrid loss functions could guide the generator toward more realistic outputs. Another promising direction involves the use of perceptual or frequency-based discriminators to better capture subtle anatomical patterns present in real medical images.

To contextualize our findings, we compare them with the state-of-the-art. Several recent works have attempted to detect GAN-generated medical images with varying levels of success. In our work, although the classifiers achieved near-perfect performance on many datasets, similarly high accuracy has also been reported in previous studies that used handcrafted features for GAN detection. This aligns with our results, suggesting that the image filters and classifiers used effectively leverage such discrepancies. Moreover, recent benchmarks such as MedFake or those inspired by the DeepFake Detection Challenge (DFDC) [20] show that classical machine learning approaches combined with compression-aware or frequency-based features can yield comparable accuracy in distinguishing real from synthetic medical images. Therefore, although our results may appear ideal in several scenarios, they are not entirely unprecedented in the literature and align with a broader trend observed in state-of-the-art GAN detection tasks within the medical domain.

## 5. Conclusions and Future Work

Considering the possibility to generate synthetic images offered by GAN and the need of huge datasets to perform biomedical research, in this paper, we proposed a method aimed to discriminate between real bioimages and fake ones (generated by exploiting a DCGAN). We proposed six different case studies, aimed to evaluate the proposed context on different bioimage types and experimental conditions. The results highlight varying levels of success across datasets. For the BLOOD and Breast datasets, classifier accuracy declines over time, suggesting that the synthetic images fail to capture the complexity of the real data. Conversely, for the Tissue and Organ_AMNIST datasets, accuracy steadily improves, indicating that the DCGAN generates increasingly realistic synthetic images. The Organ_SMNIST and Organ_CMNIST datasets exhibit “V”-shaped trends, reflecting initial challenges in adaptation followed by partial recovery, likely due to improved synthetic image quality in later epochs. Overall, the method performs inconsistently across datasets, with the Tissue and Organ_AMNIST datasets showing the most promising results.

As future work, we will investigate about the adoption of different GAN architectures (for instance, the Wasserstein Generative Adversarial Network developed to improve the learning stability) with the aim to address the inconsistencies observed across datasets by enhancing the quality of synthetic images generated by the GAN. For instance, one possible direction is to refine the architecture or loss functions of the DCGAN to better capture the complexity and variability of datasets like BLOOD and Breast.

## Figures and Tables

**Table 1:** Generator model architecture.

#	Layer (Type)	Output Shape	Params
1	Dense	(None, 12,544)	1,266,944
2	BatchNormalization	(None, 12,544)	50,176
3	ReLU	(None, 12,544)	0
4	Reshape	(None, 7, 7, 256)	0
5	Conv2DTranspose	(None, 14, 14, 128)	819,328
6	BatchNormalization	(None, 14, 14, 128)	512
7	ReLU	(None, 14, 14, 128)	0
8	Conv2DTranspose	(None, 28, 28, 64)	204,864
9	BatchNormalization	(None, 28, 28, 64)	256
10	ReLU	(None, 28, 28, 64)	0
11	Conv2D	(None, 28, 28, 1)	1601

**Table 2:** Discriminator model architecture.

#	Layer (Type)	Output Shape	Params
1	Conv2D	(None, 14, 14, 64)	1664
2	BatchNormalization	(None, 14, 14, 64)	256
3	LeakyReLU	(None, 14, 14, 64)	0
4	Conv2D	(None, 7, 7, 128)	204,928
5	BatchNormalization	(None, 7, 7, 128)	512
6	LeakyReLU	(None, 7, 7, 128)	0
7	Flatten	(None, 6272)	0
8	Dropout	(None, 6272)	0
9	Dense	(None, 1)	6273

**Table 3:** Experimental results obtained from the six datasets used in this study.

Dataset	Epoch	Precision	Recall	F-Measure	Accuracy
Blood_MNIST	0	1	1	1	1
Blood_MNIST	24	1	1	1	0.99
Blood_MNIST	49	0.99	0.99	0.99	0.99
Breast_MNIST	0	1	1	1	1
Breast_MNIST	24	1	1	1	1
Breast_MNIST	49	1	1	1	1
Tissue_MNIST	0	1	1	1	1
Tissue_MNIST	24	1	1	1	1
Tissue_MNIST	49	1	1	1	1
Organ_SMNIST	0	1	1	1	1
Organ_SMNIST	24	1	1	1	1
Organ_SMNIST	49	1	1	1	1
Organ_CMNIST	0	1	1	1	1
Organ_CMNIST	24	1	1	1	1
Organ_CMNIST	49	1	1	0.99	0.99
Organ_AMNIST	0	1	1	0.99	0.99
Organ_AMNIST	24	1	1	1	1
Organ_AMNIST	49	1	1	1	1

## Data Availability

The datasets exploited are freely available from the MedMNIST, a repository of biomedical image classification resources, freely available for research purposes at the following url https://medmnist.com/, 24 June 2025.
